# Gross‐dependent lower limb lymphoedema

**DOI:** 10.1002/ccr3.795

**Published:** 2017-01-19

**Authors:** Mairead Marion Hennessy, Gavin Connor O'Brien

**Affiliations:** ^1^ Department of General & Vascular SurgeryMercy University HospitalCorkIreland

**Keywords:** Bilateral, chronic, lower limb swelling, lymphoedema, treatment

## Abstract

Gross‐dependent lower limb lymphoedema is an unusual condition which can be painful particularly if ulceration occurs. Focused history and clinical examination in addition to appropriate radiological investigation aid in the diagnosis. It is difficult to treat and requires a multidisciplinary team including vascular surgeons, dermatologists and clinical nurse specialists. The primary treatment option is compression bandaging.

## Introduction

A definite cause for lower limb swelling must be made, and a careful history and clinical examination in addition to appropriate confirmatory tests are essential. Bilateral swelling is usually due to systemic causes, while unilateral swelling is usually due to localized causes. However, bilateral leg swelling can be more obvious in one leg than the other and can therefore be mistaken as unilateral leg swelling.

This report aims to describe a presentation of bilateral lower limb swelling and describe differential diagnoses and the treatment options.

**Figure 1:**
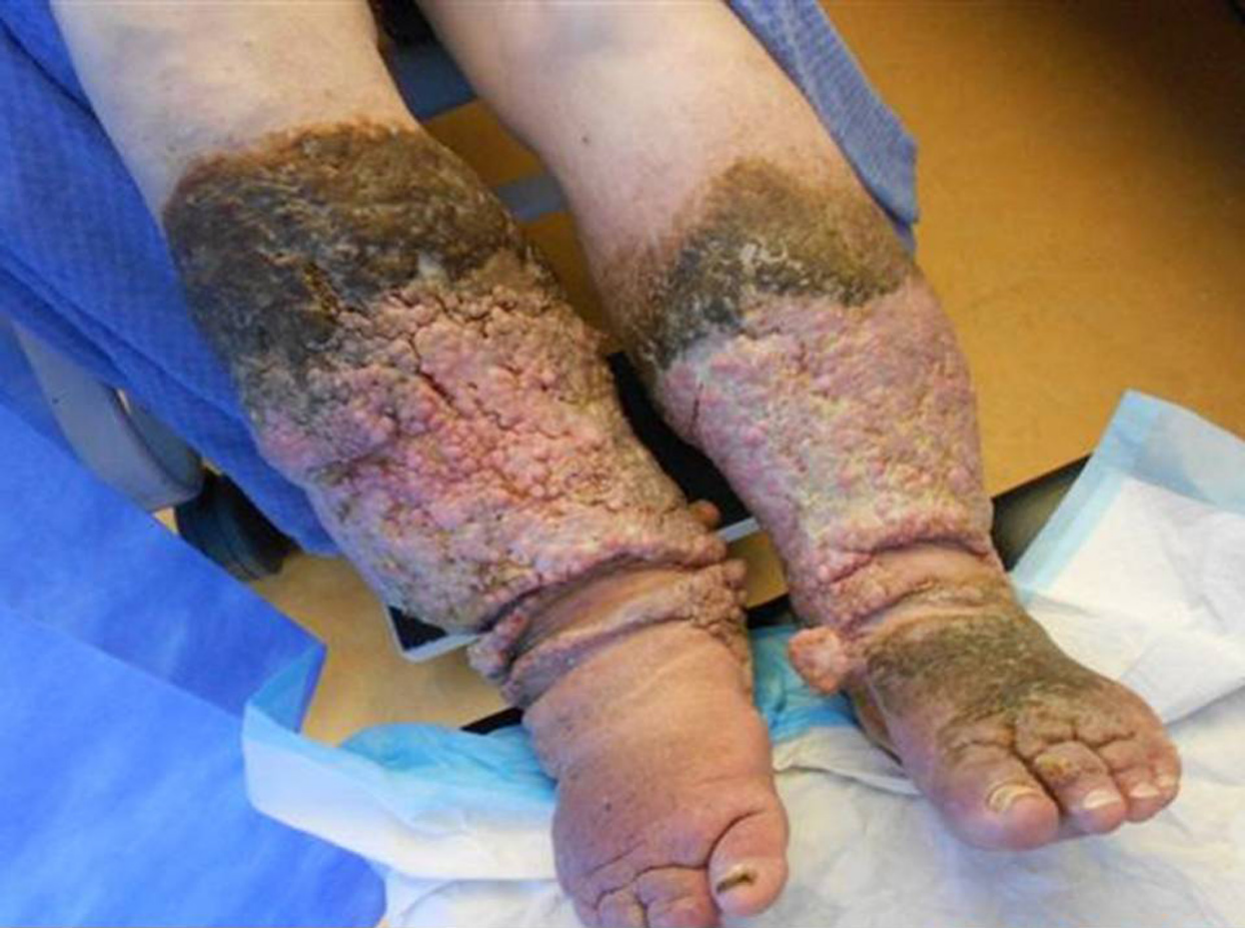
Gross dependent lower limb lymphoedema.

This is the case of a 76‐year‐old gentleman with mental illness. He had severe agoraphobia and a history of hoarding. He had been sitting and sleeping in his armchair surrounded with debris for about 3 years. He had never left the house, by his own admission. Comorbidities included obesity, hypertension, dyslipidemia, and a new diagnosis of non‐insulin‐dependent diabetes mellitus. Biometric analysis included weight 123 kg, height 1.78 m, body mass index 38.8. He developed chronic lower limb swelling as pictured (Fig. 1).

This patient's mental health led to the development of the bilateral lower limb swelling.

**Figure 2:**
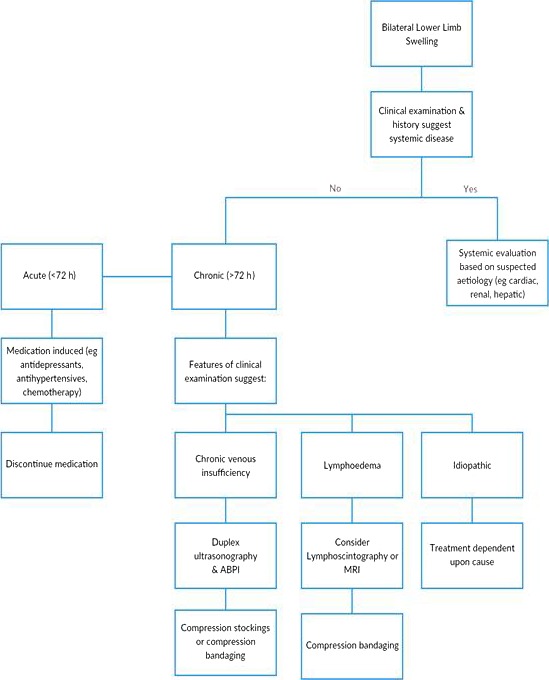
Management algorithm for lymphoedema.

Questions to consider when a patient presents with bilateral lower limb swelling include the following (Fig. 2):

1. Signs & symptoms
  - Pain
  - Heaviness
  - Pruritis
  - Erythema
  - Infection
  - Ulcers
2. What is the differential diagnosis for bilateral swollen lower limbs?
  - Congestive cardiac failure
  - Chronic venous insufficiency
  - Acute kidney injury
  - Chronic kidney disease
  - Pelvic or para aortic lymphadenectomy
  - Nephrotic syndrome
  - Cirrhosis
  - Medications
  - Pregnancy
  - Deep vein thrombosis (DVT) – usually unilateral
  - Thrombophlebitis – usually unilateral
3. What investigations would you perform? One could consider:
  - Lymphoscintigraphy
  - Ultrasound Doppler
  - Ankle‐brachial pressure index (ABPI)
  - Consider CT or MR angiogram if concerned about arterial perfusion
4. Results This patient had an ultrasound Doppler performed which did not show any venous incompetence.
5. What is the diagnosis in this case? Bilateral lower limb lymphoedema
6. How would you manage this patient?
  - Elevation and compression bandaging (pulses present)
  - Other treatment options which could be considered are as follows: Intermittent pneumatic compression boots Lymphatic venous anastomosis
7. What are the potential complications of chronic lower limb swelling?
  - Pain
  - Difficulty mobilizing
  - Pruritis
  - Infection
  - Risk of developing skin ulcers
  - Scarring
  - Reduced circulation

This patient was admitted to long‐term care upon discharge from the acute hospital. The lymphoedema improved, but he continues to wear compression bandaging. This is expected to be lifelong.

## Literature

Major papers on this subject are uncommon. Much of the literature focuses on the development of lymphoedema postoncologial surgery, particularly breast surgery. Regarding dependent lymphoedema, the literature mainly consists of retrospective reviews. I could not find one major paper. The Position Statement of the National Lymphoedema Network (updated: February 2011) did provide some insightful information about lymphoedema. O'Malley et al. 1. described clearly obesity‐related chronic lymphoedema‐like swelling and physical function. This has some relevance to our case given our patient's elevated BMI. The diagnosis and its subsequent management are both challenging. This has not improved since Browne's 2 paper in 1986. Compression therapy 3, 4 is the primary treatment option. Negative pressure compression 5, 6 does have a role. Lymphovenous anastomosis 7, 8 can improve signs and symptoms but this is only valuable in early disease. Patients’ quality of life [9, 10] is profoundly effected by lymphoedema. Simple tasks we all take for granted can be extremely challenging for those with lymphoedema.

## Authorship

MMH: conceived of the idea, researched the topic and is the primary author. GCO'B: proof‐read the report and provided advice and suggestions that enhanced the report. All authors were involved in the writing of this case report and its final approval.

## Conflict of Interest

None declared.
